# Online information analysis on pancreatic cancer in Korea using structural topic model

**DOI:** 10.1038/s41598-022-14506-1

**Published:** 2022-06-23

**Authors:** Wonkwang Jo, Yeol Kim, Minji Seo, Nayoung Lee, Junli Park

**Affiliations:** 1 Department of Public Health Sciences, Graduate School of Public Health, Seoul National University, Seoul, Republic of Korea; 2 National Cancer Control Institute, National Cancer Center, Goyang, Republic of Korea; 3 Department of Family Medicine, Myongji Hospital, Goyang, Republic of Korea; 4 Institute of Health and Environment, Seoul National University, Seoul, Republic of Korea

**Keywords:** Disease prevention, Health policy, Public health, Cancer

## Abstract

Inappropriate information on a deadly and rare disease can make people vulnerable to problematic decisions, leading to irreversible bad outcomes. This study explored online information exchanges on pancreatic cancer. We collected 35,596 questions and 83,888 answers related to pancreatic cancer from January 1, 2003 to May 31, 2020, from Naver, the most popular Korean web portal. We also collected 8495 news articles related to pancreatic cancer during the same period. The study methods employed were structural topic modeling, keyword frequency analysis, and qualitative coding of medical professionals. The number of questions and news articles increased over time. In Naver’s questions, topics on symptoms and diagnostic tests regarding pancreatic cancer increased in proportion. The news topics on new technologies related to pancreatic cancer from various companies increased as well. The use of words related to back pain—which is not an important early symptom in pancreatic cancer—and biomarker tests using blood increased over time in Naver’s questions. Based on 100 question samples related to symptoms and diagnostic tests and an analysis of the threaded answers’ appropriateness, there was considerable misinformation and commercialized information in both categories.

## Introduction

An increasing number of people are retrieving medical information on the Internet. Such information has a significant impact on people’s health behaviors^1–4^. On the bright side, information retrieval through the Internet has increased easy access to medical information for the public. However, several negative side effects have been reported. Health behavior can be influenced in the wrong direction by misinformation^5,6^.

The present study analyzed information exchange related to pancreatic cancer on the Internet. We focused on pancreatic cancer for two reasons. First, pancreatic cancer is lethal; thus, misinformation can have a significant negative impact on patients. The mortality rate of pancreatic cancer is extremely high, and early diagnosis is difficult^7^. The five-year survival rate of pancreatic cancer is approximately 10 percent in the United States (2010–2016) and 12.6 percent in South Korea (2014–2018)^8,9^. The high fatality rate can create a significant fear of pancreatic cancer among people and the fear increases the likelihood that people might accept inappropriate information^10–13^.

Second, the incidence rate of pancreatic cancer is low compared to that of other cancers, making the supply of adequate information about pancreatic cancer insufficient. In 2020, pancreatic cancer was the 12th most common cancer in the world^14^, meaning it does not always receive wide social attention, and there is a limited amount of appropriate medical expert information^15,16^. Therefore, non-experts could become interested in pancreatic cancer not because of medical experts' warnings but because of families’ or acquaintances’ experience of the disease, creating relatively little opportunity to obtain appropriate information from medical experts.

In short, online information exchange for pancreatic cancer must be monitored and measured, considering the fear of the disease due to its high fatality risk and the limited availability of adequate information on it^11,13,15^. To maximize the positive consequences and minimize the negative results of people's online search for medical information on pancreatic cancer, we need to understand the current situation of the online information and to evaluate whether adequate information is available in the online space. Analyzing online information exchanges related to pancreatic cancer, this study contributes to solving health problems associated with it, such as delayed diagnosis of pancreatic cancer, exposure to misinformation and incorrect treatment, or over-care.

Many studies have investigated the accuracy of medical information distributed over the Internet. However, most studies have focused on infectious diseases that attract significant public attention over a short time. MERS and COVID-19 are good examples^17–20^. Their importance is evident; however, deadly diseases targeting a small population, such as pancreatic cancer, also present an urgent need to measure online-based information exchanges regarding them. Several studies have reported that online information related to pancreatic cancer is often inaccurate or incomplete^15,16^. If online information on fatal diseases, such as pancreatic cancer, is insufficient, the effects of this can also be substantial. Examining the information exchange on pancreatic cancer over the long term is as important as analyzing information about new infectious diseases.

In this study, we focused on information exchange on pancreatic cancer symptoms and examination over the Korean Internet. Symptoms and examinations are topics that laymen might look for on the Internet before they visit professional healthcare providers. Therefore, information exchange on these two topics is likely to affect the general public’s health behaviors without health professionals’ practice or intervention. By delving into both topics, we estimated the pattern and effect of pancreatic cancer-related information exchange on the Internet, which is relatively free from medical expert intervention. We chose the Korean Internet because the Internet penetration rate and usage rate in Korea is very high^21^ and ample data from active information exchange on pancreatic cancer among people is anticipated. We also took into consideration the fact that the authors are familiar with the Korean medical environment and culture, and can therefore perform a more accurate analysis of Korean data.

Furthermore, we explored official media coverage, an influential factor that affects such information retrieval activities on the Internet. The exchange of information online is not an independent and isolated action; but rather, one that is dependent on various social components, such as media articles^22,23^. In other words, official media articles unconsciously affect people’s interests, thus generating information search. We examined the correlation of proportion change between the topic of reports on pancreatic cancer in the official press and the topic of voluntary information exchange on the Internet.

A summary of the research questions is as follows:

1. What are the characteristics of non-experts’ search activities over the Internet for pancreatic cancer symptoms and testing methods?
2. How do these features change over time?
3. How appropriate is information on pancreatic cancer available online?
4. What kind of relationship exists between pancreatic cancer articles in the official press and Internet information search?

## Materials and methods

### Materials

This study collected and analyzed data from the Naver Q&A forum known as “Jisikin” (in Korean, ‘지식인’, meaning intellectual), a service that has launched in 2002. There, users are free to post and answer various questions. We assumed that questions and answers related to the symptoms or examinations of pancreatic cancer would be the most suitable material for online information exchange. There are two reasons for this. First, the “Jisikin” online Q&A forum is in Naver, which is the largest and most active internet portal service in South Korea^24–26^, making it representative. Second, the data are in the form of questions and answers, making it suitable for identifying what people are curious about and determining the appropriateness of the information. Among the posts that people voluntarily write on the Internet, those in the form of questions result from the authors’ conscious efforts to help readers understand their intentions. Therefore, it is easier to understand the authors’ focus and interests from posts that are in the form of a question rather than other types of posts, such as Twitter posts. Tweet posts are often written simply to express users’ feelings about a particular object (e.g., cancer sucks!!!!), which makes it difficult to know what information users are curious about and what information they receive. Naver Q&A forum posts are therefore more suitable for analyzing Internet information exchanges because they are in the form of questions and answers.

**Figure 1:**
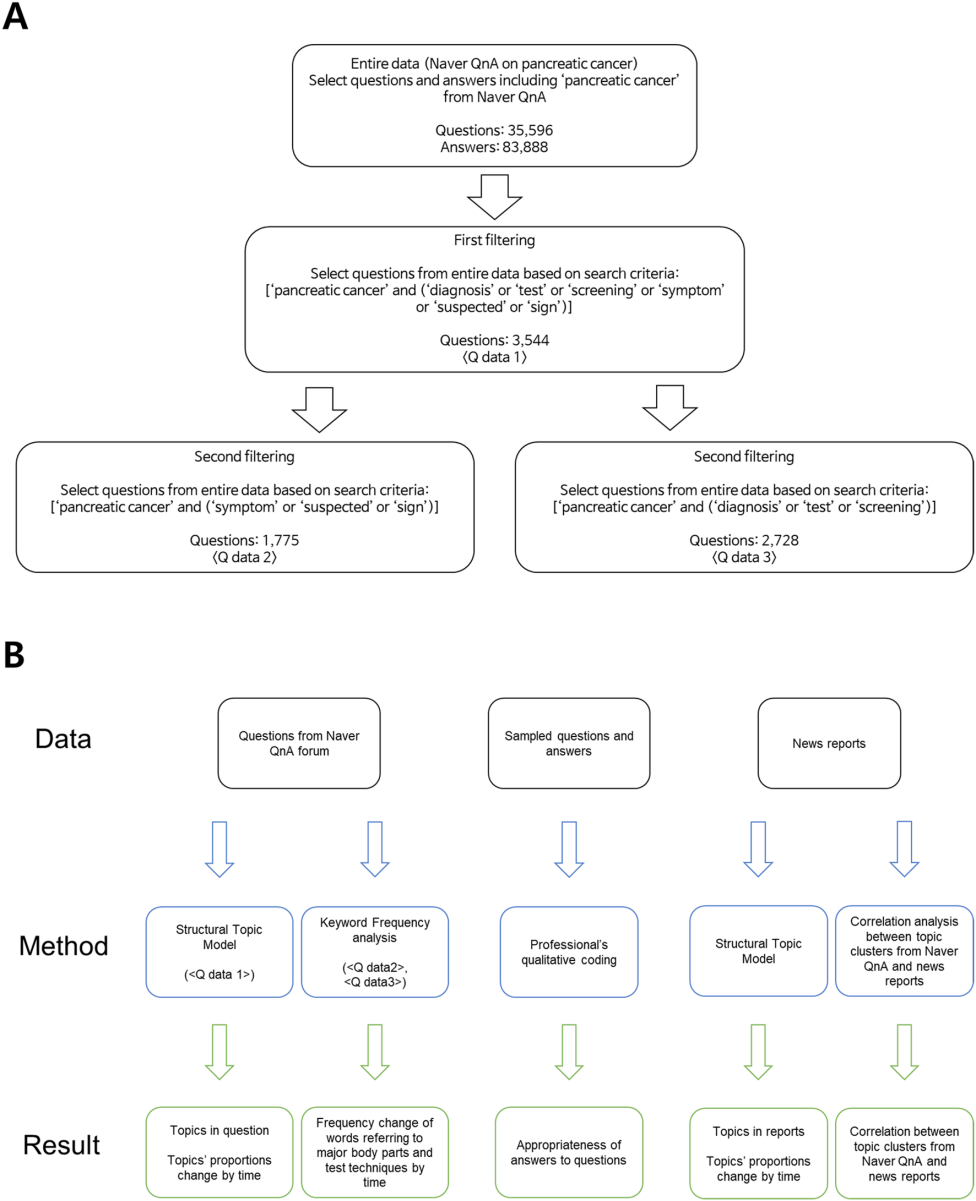
Naver Q&A data search process and overall structure of analyses (**A**) The actual data search was made using the Korean words corresponding to the English words written above. The Korean translation of each term is as follows—pancreatic cancer: 췌장암, diagnosis: 진단, test: 검사, screening: 검진, symptom: 증상, 증세, suspected: 의심, sign: 징후. Two Korean words (증상, 증세) correspond to “symptom,” so we used both words. As can be inferred from the search conditions, some questions correspond to < Q data 2 > and < Q data 3 > simultaneously. (**B**) The overall structure of analyses in this paper.

We collected Naver Q&A data through the procedures given below to identify the information that people have explored on pancreatic cancer by analyzing their questions, especially what they ask about testing and symptoms, and the answers given. Naver’s Jisikin search system does not support various Boolean operators such as “or” for data searching. Thus, we collected the entire data, including the word ‘췌장암’ (in English, pancreatic cancer), in the answers or questions. Subsequently, we extracted questions and answers about symptoms and examinations using more detailed search conditions from this dataset (see Fig. 1A). The data collection period was from January 1, 2003, to May 31, 2020.

First, we extracted questions that people raised about two topics: pancreatic cancer symptoms and examinations. The search conditions are shown in Fig. 1A (first filtering). We identified the topics people paid attention to by applying a structural topic model (STM)^27^ to the data (Q data 1), which is explained later in the study. Questions about symptoms (Q data 2) and tests (Q data 3) were collected separately (second filtering). The search criteria are shown in Fig. 1A. To see the changes in keyword frequency for each symptom and examination, we collected only the questions about symptoms and only those on examination.

We performed basic preprocessing. First, we eliminated duplicate questions. Since Jisikin is run with the voluntary participation of users, duplicate questions may be present. Occasionally, a user uploads the same question more than once differing only by one or two letters. We based the removal of duplicate questions on whether the first or last 50 elements of the questions were identical, where ‘elements’ means anything that occupies positions in character strings, such as letters, spaces, and punctuation. Additionally, the Korean word for back (body part) is “등,” which is a homonym, meaning also “other.” Thus, we tried to distinguish words that reference body parts among those having the same form (i.e., ‘등’) in the questions. When other body parts appear near “등” or words related to the back appear, we classify “등” with a separate symbol to indicate that the “등” is referencing the back.

We also collected official media articles related to pancreatic cancer to analyze the relationship between people’s autonomous information-seeking activities and official media coverage. We utilized a database named “BIGKinds,” which is run by Korea Press Foundation and enables users to collect Korean media reports (www.bigkinds.or.kr). We collected 8,495 articles from 17 famous media sources (a list of media sources is shown in Supplementary Table S1). The same collection period as that of Naver Q&A (January 1, 2003, to May 31, 2020) was used. Duplicate or insignificant articles were deleted based on two criteria. The first was whether BIGKinds categorized the article as “exception” or “duplication.” For each article, BIGKinds provided information on whether it was estimated as a duplicated or insignificant article. Because the BIGKinds system automatically collects articles, sometimes the same article can be collected several times. Further, it could include some insignificant articles because the article is just an obituary or a simple photo. BIGKinds excludes such articles from the analysis. The second criterion was whether the first or last 50 elements of the articles’ keywords were identical. A total of 8,495 articles remained after deleting duplicate or insignificant articles.

### Methods

This study aimed to analyze what kind of topics exist in the questions related to pancreatic cancer, how the proportion of the topics change over time, and how appropriate and accurate the information is that people acquire. We utilized three methods: Structural topic model (STM), keyword frequency analysis, and qualitative coding. The overall structure of the analyses is described in Fig. 1B.

#### Structural topic model

STM is a topic modeling method^27^. It is almost impossible for researchers to read thousands of questions and articles directly and estimate the topics in them accurately and objectively. Topic modeling methods utilize statistical techniques to estimate topics from numerous documents automatically. Therefore, they are well suited to our aim of assessing subjects from many documents.

We used STM among various topic modeling methods. This is because our purpose is to estimate intuitively understandable topics from many documents and to estimate how the estimated topics fluctuate over time in proportion. Topic modeling is a generic name for various techniques such as latent semantic analysis (LSA)^28^, probabilistic latent semantic analysis (PLSA)^29^, and latent Dirichlet allocation (LDA)^30^. We decided to utilize STM considering the advantages and disadvantages of various topic modeling techniques.

LSA is the earliest form of topic modeling. It applies truncated singular value decomposition (SVD) to a term-document matrix to estimate the factors that summarize the association of terms and documents. If the term-document matrix’s dimension is M × N and an analyst decides to estimate K factors, the term-document matrix is approximated through SVD with a product of three matrices: the M × K, K × K dimension, and K × N dimensions. The first M × K matrix shows the terms’ association with K factors, and the third K × N matrix shows the documents’ associations with K factors. We can interpret these factors as topics because they summarize the information on the associations of terms and documents. However, the factors of LSA are more difficult to intuitively understand than those of PLSA and LDA. The other two methods (PLSA and LDA) are designed to give the topic the meaning of the probability distribution, so the association between topic and word or topic and document in the methods has a value between 0 and 1. In contrast, the two matrices derived by LSA can contain negative values. Therefore, the result cannot be interpreted as a probability, and is difficult to understand intuitively. Because of these attributes, researchers proposing LSA stated that the factor estimated by LSA is not for verbal description^28^.

PLSA introduces the concept of stochastic occurrence processes, allowing the topic to be understood as the probability distribution over words and the document to be understood as the distribution of topics^29^. Therefore, the results of PLSA are better than those of LSA from the perspective of interpretability. However, PLSA cannot assign topic distributions to new documents and is vulnerable to overfitting^30^. LDA uses Bayesian statistics to overcome these difficulties and utilizes the Dirichlet distribution as a prior distribution to overcome the problems of PLSA and provide a consistent process for generating documents. STM provides useful functions beyond LDA while maintaining the advantages of the LDA. In addition to simply estimating topics and document-specific topic distributions, STM can infer the effect of metadata of each document on the proportion of each topic. Metadata refers to the information about each document, like the document’s publication time, author, and publisher. It is possible that various metadata are associated with the topic proportion. For example, there may be an increase in the proportion of specific topics over time. That is, publication time and specific topic proportions may be positively correlated. STM helps identify and analyze the existence of such relationships. We used STM to estimate the key topics present in questions and media articles on pancreatic cancer and the change in the proportions of the key topics over publication year^30–34^.

The following is a brief description of how the STM works. STM estimates multiple word probability distributions that describe large volumes of documents well. In STM, “Topic” refers to the word probability distribution. Two assumptions are required for this process to be feasible. The first assumption is that a document is a bag of words. In other words, STM considers only the type and frequency of words in a document, ignoring the order of their appearance or the structure of sentences. The second assumption is that the text is generated from the probability distribution of words. For example, suppose a probability distribution of words as follows: [Corona - 0.1, Virus - 0.1, Quarantine - 0.05, Vaccine - 0.03, …]. It is sorted list of words by probability, so the “Corona” and “Virus” have highest probability in this probability distribution. If words are extracted based on this probability distribution, there will be many instances of “Corona” or “Virus” because they have a high probability. STM, based on these two assumptions, infers which word probability distributions (topics) are best for producing given documents.

In STM, this estimated word probability distribution is called “topic” because the core information of the topic exists within the word probability distribution. Let us consider how we refer to topics in real-language terms. This is mainly expressed by the disproportionate use of words. One topic contains important objects and concepts related to it. When the topic is expressed in a piece of writing, words pointing to the object or concept appear more often than other words. We deduce the topics in this article by observing these uneven word usages. The word probability distributions that topic modeling estimates summarize these unbalanced word usage patterns, informing us which group of words appears more often than other ones in the data. Therefore, the researcher can infer intuitive topics from the word probability distributions.

Additionally, STM estimates the relationship between document metadata and the proportion of topics. The extracted topics do not all have the same proportion in documents, but they exist based on a unique distribution per document. For example, one document may consist of 40% of Topic 1 and 60% of Topic 2, while another document may contain 90% of Topic 1, and 10% of Topic 2. Another essential inference from STM is the estimation of the topic distribution in each document. By the way, topics’ proportion may vary according to the metadata of each document, such as the type of media or publication time. For example, documents produced in the past may contain a higher proportion of specific topics. STM can estimate this relationship, that is, the relationship between the metadata of a document and the topic proportion in the document. This is a key function of STM. We analyzed how the expected proportion of topics in documents varies over time, assuming that the question and news article’s publication year are associated with the proportion of topics from them.

The topic interpretation process was as follows: since a topic from STM is a probability distribution over words, it includes critical information about the topic but requires researchers to use this information to interpret and label the topic. Hence, we named each topic to be understandable to humans, utilizing two kinds of information. First, we identified important words for each topic. Two criteria determined the important words in each topic: the probability of words and the FREX score of words in the corresponding topic. FREX is an indicator that considers both the frequency of words and exclusivity between topics^33^, which prevents errors that can occur when considering probability alone. For example, if a word has a high probability in all topics, the word is less valuable in interpreting a particular topic. The word’s FREX score would be low, even though the probability of the word is high. We utilized the top 15 words based on probability and the top 15 words based on FREX in each topic to interpret each topic. Second, we identified a real document with a high proportion of a particular topic. As noted earlier, the STM estimates the distribution of topics per document. Thus, a document with a high percentage of a particular topic can be seen as expressing the topic in real human sentences. This is a valuable reference for topic interpretation. We extracted the top 10 documents per topic and utilized them for interpretation. Thereafter, we named each topic using this information through a consensus between the authors.

Finally, we only utilized nouns for STM in Naver Q&A. We referred to Martins and Johnson’s research showing that limiting the analysis to nouns could improve topic coherence^35^. As for media reports, we utilized keywords that BIGKinds provided. BIGKinds explains that the keywords they provide for each article are nouns appearing in the article^36^.

#### Keyword frequency analysis

We analyzed how essential keywords related to pancreatic cancer symptoms and examinations vary over time by observing the keyword frequency. That is, we calculated the number of appearances of the keywords in each year using R (version 4.1.0). The disadvantage of topic modeling, including STM, is that researchers cannot fully control the analysis focus. For example, although a researcher wants to know about the people’s suspected symptoms of pancreatic cancer, the researcher cannot intentionally estimate the related topic, but rather only interpret the topics estimated by the statistical model. Therefore, it is difficult to explore the issues that researchers are interested in. To compensate for these limitations, we attempted to identify important words in advance and analyze how the relative frequency of these words changes over time. Naturally, an object is frequently used when it receives extensive attention; therefore, we can track changes in people’s interests from the frequency with which words are used in questions.

First, we selected words referring to major body parts and analyzed how their frequency varied over time in questions related to pancreatic cancer symptoms. In most cases, discomfort in certain body parts initiates health concerns. From a non-expert’s perspective, the body part where the person feels discomfort is crucial information. Therefore, if inaccurate information is circulated, unnecessary waste of medical resources can occur. For example, suppose back pain is considered to be an early symptom of pancreatic cancer—in such cases, those who experience back pain may misjudge that it is an early symptom of pancreatic cancer and seek medical attention. We analyzed the relative frequency changes of several words pointing to major body parts to determine which body parts people paid particular attention to when they were concerned about pancreatic cancer.

Moreover, we selected words referring to the main test techniques and observed changes in the frequency of these words in the examination-related questions. Interest in specific testing techniques for diseases is an important focus for non-specialists when inquiring about the disease. This kind of interest is also important policymaking information because it directly relates to the public use of medical services. Therefore, what testing techniques people are primarily interested in is critical information in determining the health behaviors of non-experts as well as how governments allocate health care resources in the future. To investigate this information, we analyzed the frequency changes in words pointing to basic examination techniques.

#### Professionals’ qualitative coding

Another important goal of this study was to analyze the appropriateness of pancreatic cancer-related medical information distributed on the Internet. To this end, we explored the answers to these questions. However, it is difficult to determine the appropriateness of information using topic modeling or keyword frequency analysis. Language materials in misinformation often consist of words and phrases similar to those that convey appropriate information. Thus, it is difficult to determine the appropriateness of the information by simply summarizing word usage patterns. We utilized a method in which medical professionals classify a sample of answers in each crucial area into five categories based on the appropriateness of the information: appropriate answers, unrelated answers, wrong answers, advertisements, and others. Two medical professionals, who were also the authors of this paper, mutually classified the answers. We focused on the answers to two question categories. The first category of questions was pancreatic cancer anxiety based on physical symptoms. The second was a pancreatic cancer examination. For each category, 100 representative questions were selected, and their answers were identified.

For this analysis, we created samples of questions about the suspected symptoms and examination methods of pancreatic cancer, utilizing the topics estimated by STM and topic clusters produced by Walktrap^37^. We identified a subset of topics related to suspected symptoms and diagnostic test techniques among all topics. These topics belong to cluster 3 (topics on test) and cluster 4 (topics on symptoms), which are explained in a later section. We then chose questions with a high proportion of related topics and selected the top 100 questions based on the proportion of related topics and corresponding answers. The numbers of threaded answers to the 100 questions on symptoms and tests are 147 and 135, respectively.

#### Other techniques

Several other methods have also been used. First, we utilized a held-out likelihood indicator to obtain a suitable number of topics^38^, which is a quality metric of the topic model. The number of topics to be extracted through STM is determined by the analyst; there is no established way to determine the appropriate number of topics^27^. We measured the held-out likelihood of many topic models using the same data by varying the number of topics from 10 to 80, and we selected an adequate number of topics based on the results. We did not measure held-out likelihood by increasing the number of topics to 100 or 200 beyond 80 because one of our purposes of utilizing STM was to summarize a lot of text data. Estimating too many topics violates the purpose. Thus, we think it is appropriate to set the number of topics based on held-out likelihood within a limited range.

Second, to summarize the results of STM, we utilized the Walktrap^37^ algorithm, a network community detection algorithm^39,40^. A total of 53 topics were estimated from the Jisikin questions and 75, from newspaper articles. These topics are summarized information, considering that the raw data are in thousands of documents. However, 53 or 75 topics still provide much information for humans to understand briefly. Thus, we assumed topics as nodes and a positive correlation between topics as a link between them and applied the Walktrap algorithm, whose random walk length is two, to identify a set of topics that are judged to appear together and consist of a cohesive group. For example, we can determine that topics 1, 3, and 10 frequently appear together by applying a Walktrap. We can also interpret these topic sets. The interpretation of the topic set encompasses the interpretation of the individual topics belonging to the set. In other words, we analyzed a bigger theme by finding a cohesive group of topics.

Third, simple linear regression models were utilized to determine whether the proportion of topics increased and decreased over time, and Pearson’s correlation coefficients were employed to determine whether one topic cluster proportion was associated with another. We interpreted only statistically significant ($P value\le 0.05$) coefficients.

Finally, we utilized a morpheme analyzer called Komoran^41^ to extract words and other information from natural sentences. There are many morpheme analyzers for Korean sentences; however, we chose Komoran because of its performance quality, having received an award from the National Institute of Korean language for excellent performance. We ran the Komoran morpheme analyzer using Python’s Konlpy library^42^. In the case of news articles, as we mentioned before, BIGKinds provides keywords from individual reports; thus, we utilized the keywords provided by BIGKinds.

Except for the morpheme analyzer, all the methods and visualizations were implemented using the statistical programming languages R^43^ and libraries of R. A specific list of the libraries used was as follows: tidyverse, textclean, tidytext, widy, lubridate, slam, igraph, ggraph, cowplot, psych, and stm^27,44–53^.

## Results

### Structural topic model

The number of questions related to pancreatic cancer symptoms and tests on Naver grew over time (see Supplementary Fig. S1). We estimated 53 topics from 3543 questions related to pancreatic cancer symptoms and tests. Figure 1A illustrates that the question data number is 3544 (Q data 1); however, 3543 data items were utilized because one question that consisted of very few word types was eliminated. We only utilized nouns that appeared in more than one question and questions that consisted of at least two kinds of nouns.

As previously discussed, we determined the number of suitable topics based on the held-out likelihood. As shown in Supplementary Fig. S2, we extracted 53 topics because the held-out likelihood was the highest when 53 topics were estimated.

We applied Walktrap to classify the 53 topics into related topic sets or clusters. The visualization of the results is shown in Supplementary Fig. S3. We identified four clusters or sets composed of two or more topics.

We interpreted 53 topics using a previously described procedure. The results are presented in Supplementary Table S2. The table contains the topic number, the interpretation of each topic, and the cluster number of each topic. Topic numbers are nominal numbers for distinction, and so are cluster numbers. To ensure that related topics are listed together, the topics are arranged based on the cluster number to which they belong. The interpretation of the four clusters, i.e., the four major themes we extracted, are as follows: Cluster 1: < Questions based on information and experience about pancreatic cancer > , Cluster 2: < Insurance-related topics > , Cluster 3: < Pancreatic cancer screening and examination > , Cluster 4: < Suspected pancreatic cancer that started with symptoms > .

**Figure 2:**
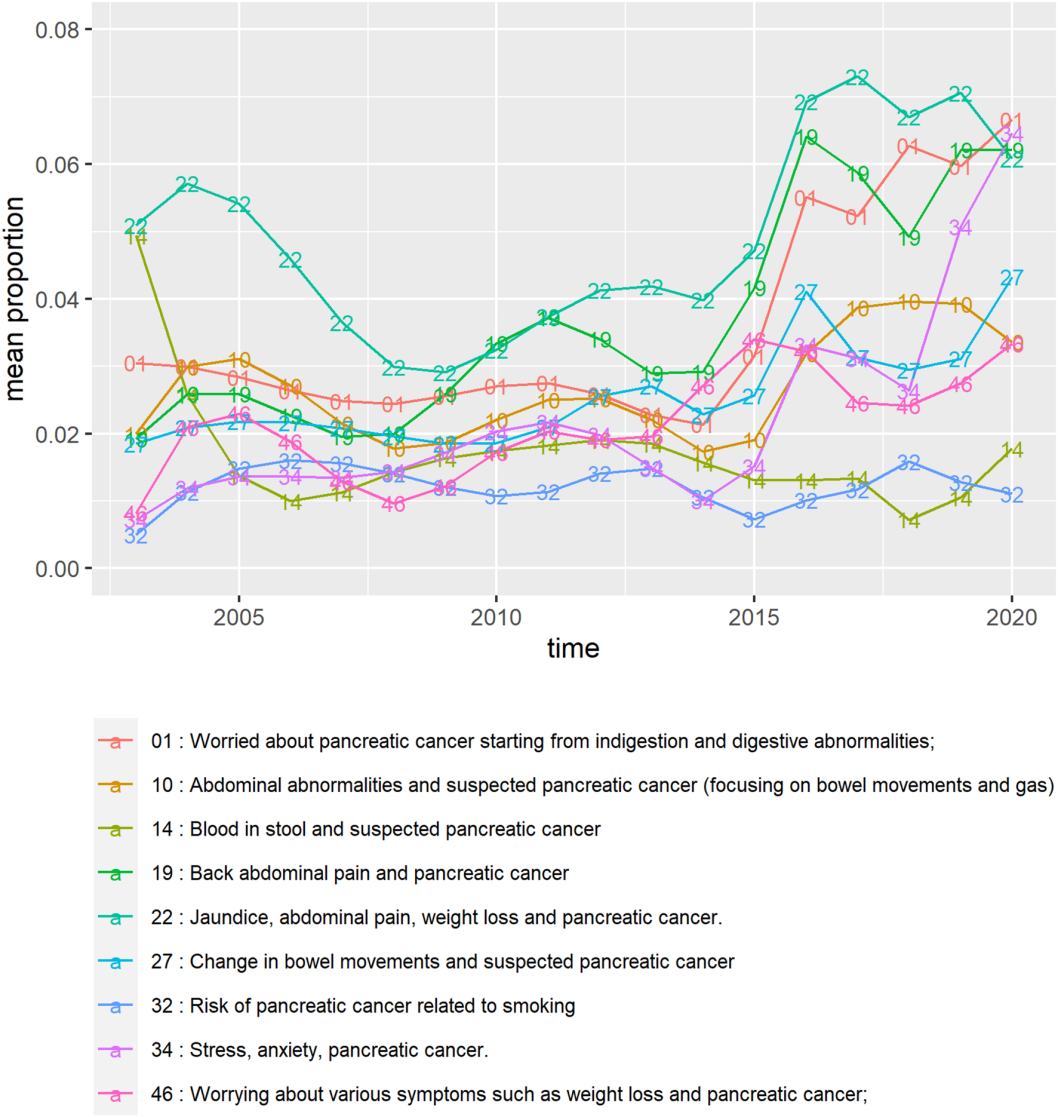
Changes in topic proportions over time regarding symptoms of pancreatic cancer. The topics in this figure belong to cluster 4—“Suspected pancreatic cancer that started with symptoms”.

We particularly noted the topics related to pancreatic cancer screening and suspected pancreatic cancer following physical symptoms. Based on the topic clusters, we paid special attention to the topics belonging to Clusters 3 and 4. We analyzed changes in the proportion of these topics over time (publication year). The changes in the proportion of suspected pancreatic cancer topics due to physical symptoms (topics in Cluster 4) are shown in Fig. 2.

Most of these topics are supposed to have been extracted from symptom-based questions asked by people without contacting a medical institution. It can be observed that the proportion of most displayed topics has increased. We made simple linear regression models with each topic proportion as a response variable and year as an explanatory variable; that is, we made nine simple linear regression models and interpreted the year coefficients. In models with seven topic (1, 10, 19, 22, 27, 34, 46) proportions as a response variable, the time variable was estimated to have a significant positive relationship with topic proportions; that is, the topics were estimated to increase over the year. Topic 14 was estimated to decrease over time. (Summary of simple linear regression models is shown in Supplementary Table S3.)

**Figure 3:**
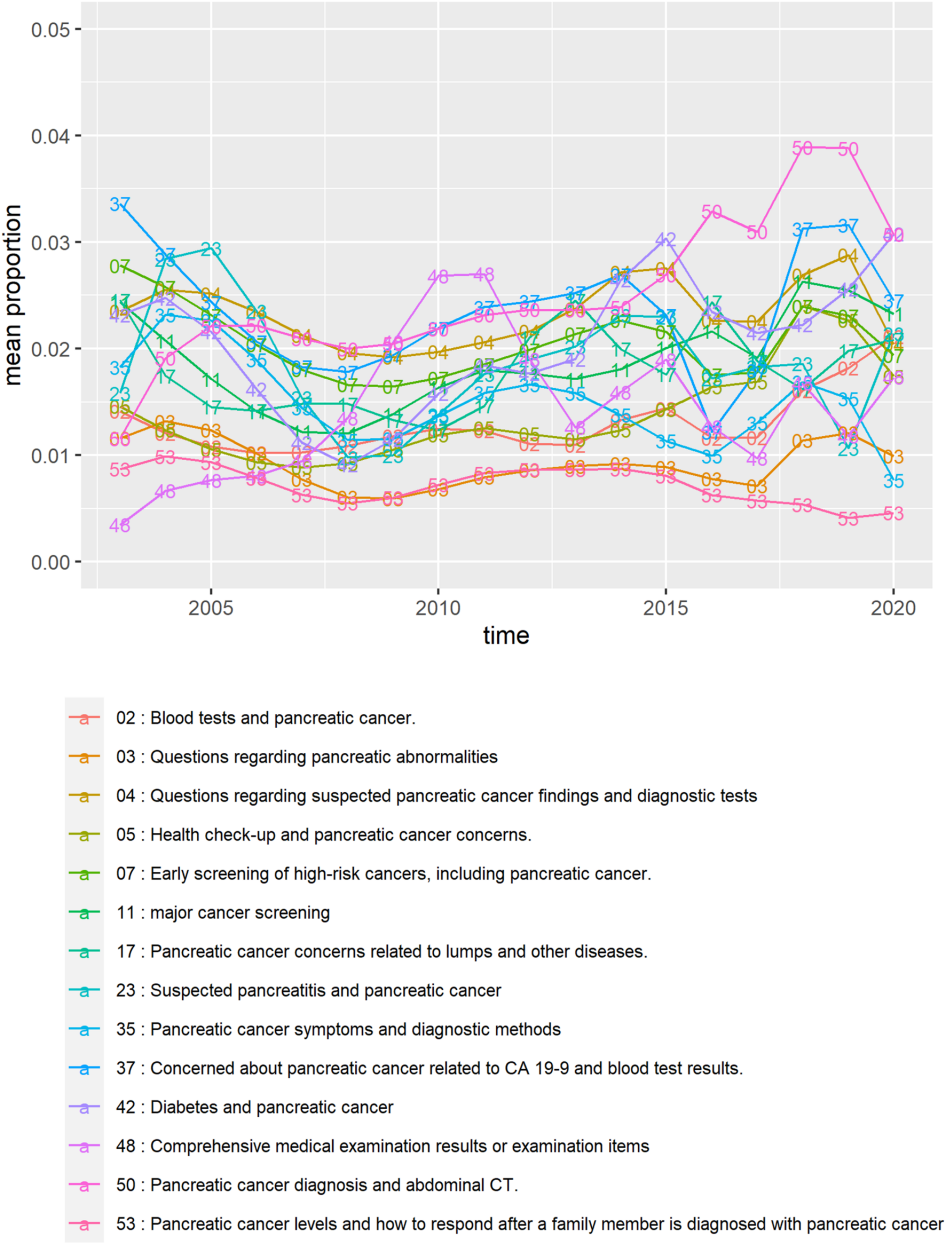
Changes in topic proportion over time regarding diagnostic tests of pancreatic cancer. The topics in this figure belong to cluster 3—“Pancreatic cancer screening and examination”.

The changes in the proportion of topics associated with pancreatic cancer tests over time are shown in Fig. 3.

In models with the five topic (2, 5, 11, 42, 50) proportions as a response variable, the time variable was estimated to have a significant positive relationship with topic proportions. Conversely, the two topics (35, 53) were estimated to decrease over time. (Summary of simple linear regression models is shown in Supplementary Table S4.)

### Keyword frequency analysis

**Figure 4:**
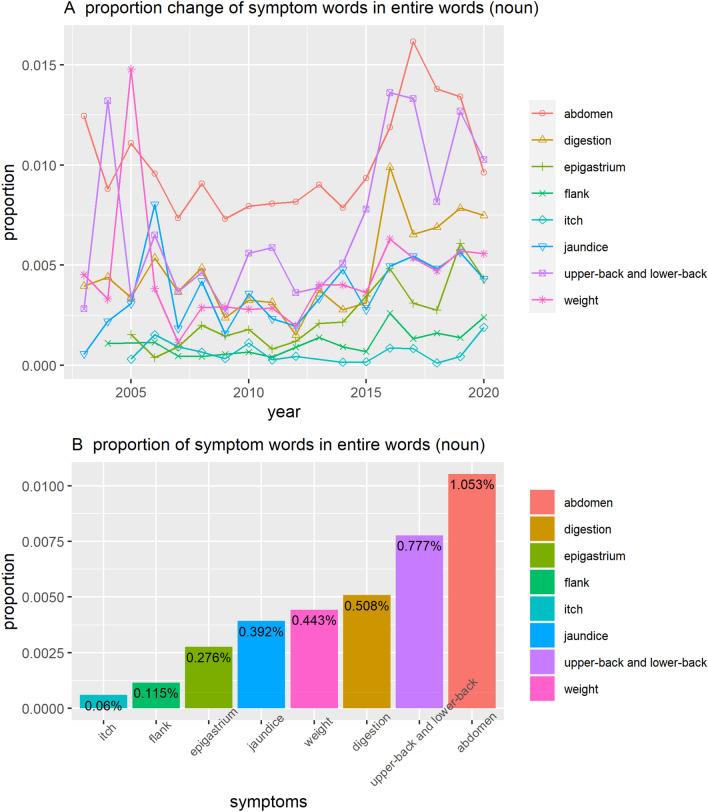
Proportion of symptom words in related questions.

The words we analyzed were related to symptoms and examination. First, we selected questions related to symptoms and analyzed the essential word frequencies related to the symptoms. The list of words we analyzed is as follows (original Korean words in parentheses): itch (가려움), upper-back (등), low-back (허리), epigastrium (명치), abdomen (복부), digestion (소화), flank (옆구리), weight (체중), and jaundice (황달). We combined the upper back and lower back frequencies because people often confuse these two regions. The analysis results are shown in Fig. 4.

Impressively, the appearance of the lower back and upper back symptom words has steadily increased, catching up to that of abdomen and taking up the highest frequency in 2020, which is surprising because pain in the lower and upper back cannot be considered an early symptom of pancreatic cancer^54^.

**Figure 5:**
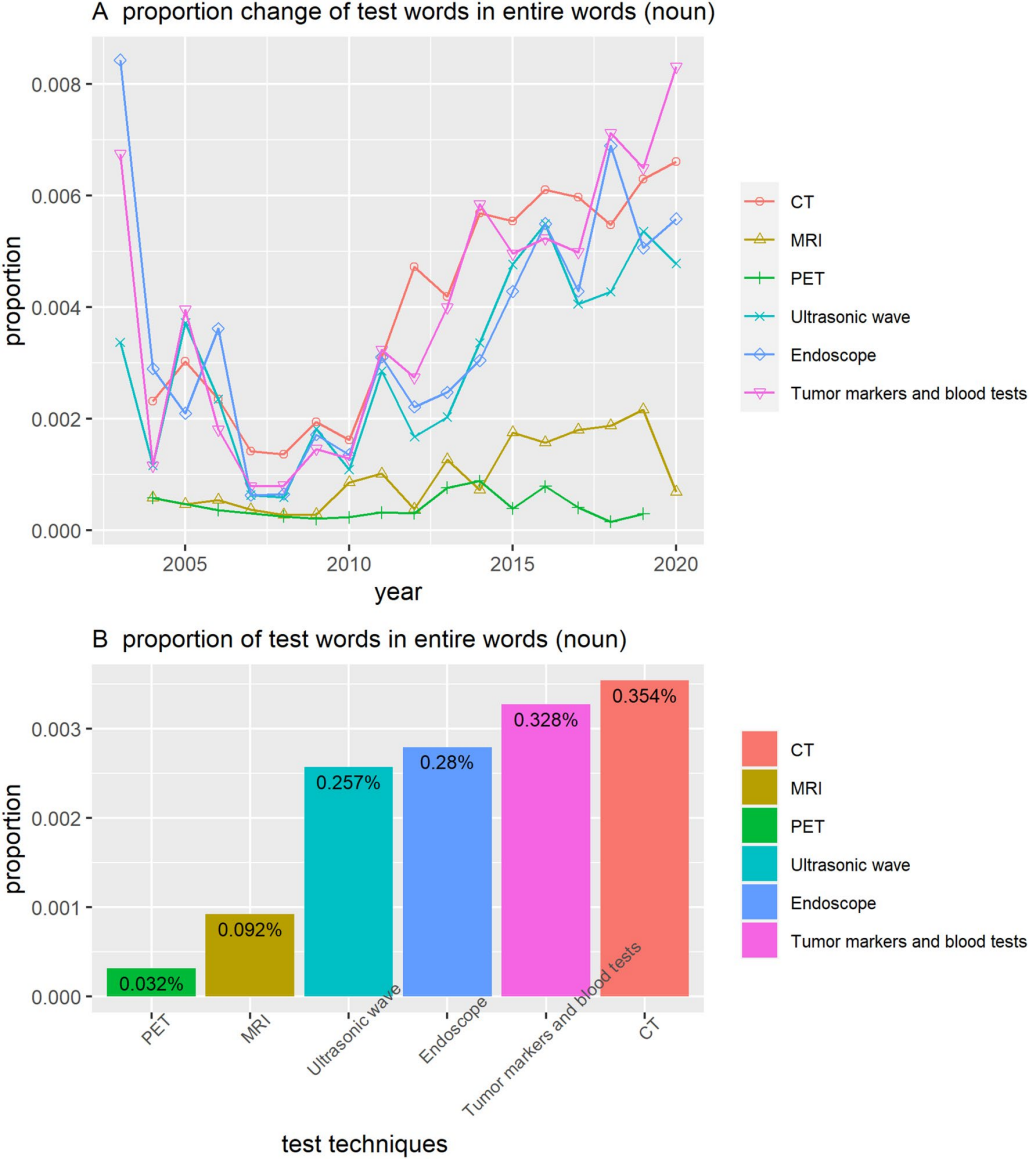
Proportion of test words in related questions. CT—Computed Tomography; MRI—Magnetic Resonance Imaging; PET—Positron Emission Tomography.

Figure 5 shows the results of a frequency analysis of the words associated with the main test method for the test questions. The list of words we analyzed is as follows: Computed Tomography (CT), Magnetic Resonance Imaging (MRI), Positron Emission Tomography (PET), Ultrasonic wave (초음파), Endoscope (내시경), and Tumor markers and blood tests (종양표지자와 혈액검사). Since people did not use the official name of the test in English, we counted the words written in parentheses behind each technique, abbreviated terms, or Korean-translated words. The frequency of "Endoscope" adds all the frequency of various endoscopy tests and “Tumor markers and blood tests” adds the frequency of various tumor markers and other blood tests.

It is notable that the proportion of words related to blood-based tests increases beyond that of CT. CT scans are more accurate than blood-based tests for pancreatic cancer screening. Thus, it is unusual for the use of words referring to blood-based tests to exceed those of CT. We speculate that this is related to accessibility or media coverage. CT and MRI are relatively expensive tests, whereas blood-based tests are not. As is discussed later in the study, many media outlets report recent research outcomes related to innovative blood tests or tumor markers, which may be reflected in these results.

### Professionals’ qualitative coding

**Figure 6:**
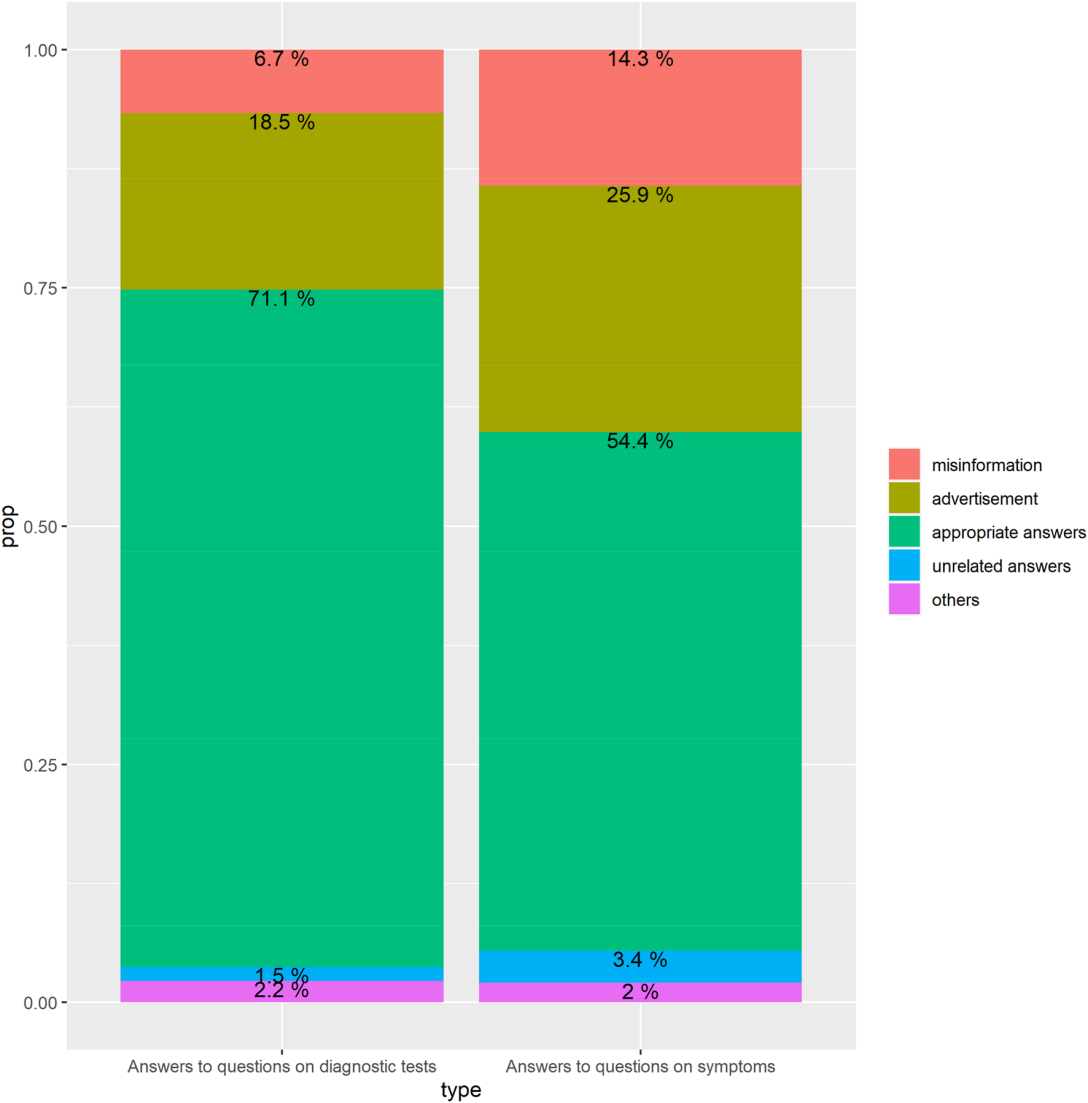
Proportion of categories of answers threaded from questions on pancreatic cancer in Naver (based on sample data).

The results of reading and classifying answers to key questions are shown in Fig. 6. By comparing the answers to the questions related to symptoms with those of the test questions, we found that there was more advertising and misinformation in the answers to the questions related to symptoms. The answers to the questions related to the diagnostic tests were also inaccurate. More than 18% of advertisements and 6% of incorrect information exist in response to questions on the tests.

### News reports analysis

There were 8,495 news items related to pancreatic cancer. Annual frequencies can be found in Supplementary Fig S4. Articles related to pancreatic cancer are increasing in number. The explosion in 2011 was believed to have been caused by the death of Steve Jobs. We checked the proportion of articles, including Jobs, in all articles for each year. In total, 48.5% of the articles appeared in 2011 alone and recorded very low results in the other years (12.8% in 2009 and less than 10% in all other years). In Korea, Yoo Sang-Chul, a famous Korean soccer player and manager, was diagnosed with pancreatic cancer, which was also an important event generating public interest in pancreatic cancer. We noted this event along with the death of Steve Jobs in Supplementary Fig S4.

We applied STM to these data and estimated 75 topics. We determined the number of topics based on the held-out likelihood. As shown in Supplementary Fig. S5, we extracted 75 topics because the held-out likelihood was the highest when 75 topics were extracted.

**Figure 7:**
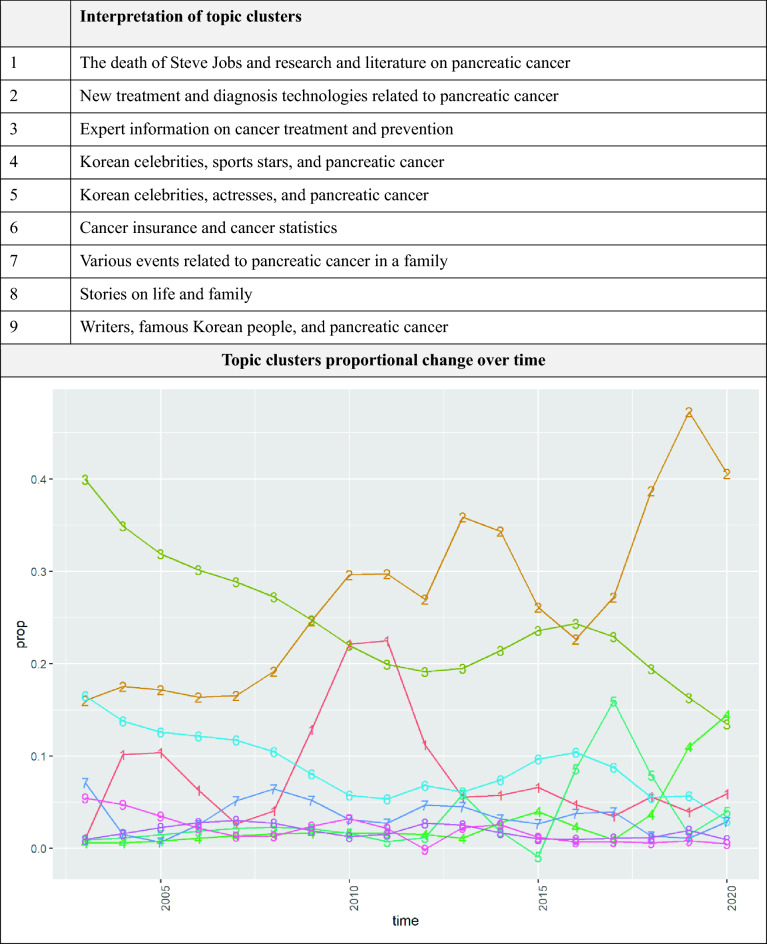
Interpretation and proportion changes of topic clusters.

As with the question analysis, we interpreted each topic while estimating a cluster of topics. Nine clusters comprising two or more topics were identified. The interpretation of the topics and each topic’s cluster number is provided in Supplementary Table S5. The interpretation of the topic clusters and their proportional changes over time (publication year) are shown in Fig. 7. We can also obtain information on the changes in the cluster proportion based on changes in the topic proportion.

The proportion of topics that provide Expert information on cancer treatment and prevention decreased (Cluster 3), and the proportion of topics on new treatment or diagnosis technologies, which are related indirectly or directly to pancreatic cancer, increased (Cluster 2). Topics belonging to Cluster 2 mainly deal with new technologies regarding the treatment and diagnosis of pancreatic cancer or cancer in general and their impact on corporate value.

We hypothesized that news of celebrity deaths from pancreatic cancer and news about new technologies related to pancreatic cancer could be associated with people’s interest in pancreatic cancer symptoms and tests. Thus, we estimated the correlation between the topic clusters corresponding to this content. That is, we analyzed how the proportion change of topic clusters extracted from the Naver Q&A forum questions correlates with the proportion change of the topic clusters extracted from the news. The results are presented in Table 1.

**Table 1:** Correlation between topic clusters.

	Question 1	Question 2	Question 3	Question 4
News 1	0.364 (0.138)	0.035 (0.889)	− 0.131 (0.605)	− 0.275 (0.269)
News 2	− 0.306 (0.216)	− 0.455 (0.058)	0.564 (0.015)	0.579 (0.012)
News 3	0.238 (0.341)	0.276 (0.268)	− 0.256 (0.305)	− 0.488 (0.04)
News 4	− 0.637 (0.004)	− 0.341 (0.167)	0.467 (0.051)	0.699 (0.001)
News 5	− 0.243 (0.331)	− 0.249 (0.318)	0.021 (0.933)	0.531 (0.023)
News 6	0.198 (0.431)	0.224 (0.372)	− 0.228 (0.364)	− 0.393 (0.107)
News 7	0.09 (0.722)	0.557 (0.016)	− 0.64 (0.004)	− 0.425 (0.078)
News 8	0.08 (0.753)	0.614 (0.007)	− 0.51 (0.031)	− 0.559 (0.016)
News 9	0.4 (0.1)	0.055 (0.83)	− 0.003 (0.992)	− 0.472 (0.048)

Cluster	Interpretation			
News 1	The death of Steve Jobs and research and literature on pancreatic cancer			
News 2	New treatment and diagnosis technologies related to pancreatic cancer			
News 3	Expert information on cancer treatment and prevention			
News 4	Korean celebrities, sports stars, and pancreatic cancer			
News 5	Korean celebrities, actresses, and pancreatic cancer			
News 6	Cancer insurance and cancer statistics			
News 7	Various events related to pancreatic cancer in a family			
News 8	Stories on life and family			
News 9	Writers, famous Korean people, and pancreatic cancer			
Question 1	Questions based on information and experience about pancreatic cancer			
Question 2	Insurance-related topics			
Question 3	Pancreatic cancer screening and examination			
Question 4	Suspected pancreatic cancer that started with symptoms			

*Note: *P* values are in parentheses.

The results show that news cluster 2 and question clusters 3 and 4 have statistically significant positive correlations. This finding suggests that news about new technologies related to pancreatic cancer may be associated with people’s interest in pancreatic cancer symptoms and tests. News cluster 4 and 5, which are about Korean celebrities and pancreatic cancer, also show significant positive correlation with question cluster 4. However, news clusters 1 and 9, which are also about famous people and pancreatic cancer, show an insignificant correlation or negative correlation with question clusters 3 and 4. News clusters 4 and 5 were about Korean celebrities and pancreatic cancer, whereas news clusters 1 and 9 were about foreign figures or other famous people, including writers. This suggests that only the pancreatic cancer experience of celebrities familiar to people (Korean celebrities in this case), not all public figure, may be associated with people's interest in pancreatic cancer symptoms and tests. However, our results are only correlations in proportion changes, and we cannot therefore comment on the causal relation, such as whether specific news topics affect public interest.

## Discussion

The number of questions related to pancreatic cancer in the Naver Q&A forum has increased, indicating the increase in demand for pancreatic cancer information in Korea. The topics proportion of suspected symptoms of pancreatic cancer increases over time. There is a noticeable point in the body's symptom areas inducing people to suspect pancreatic cancer. The use of words referring to the lower and upper back increased in questions related to the symptoms of pancreatic cancer. The proportion of topics related to diagnostic tests also increased slowly. The frequency of words referring to biomarker tests using blood increased gradually in questions related to diagnostic tests for pancreatic cancer. Overall, the number of news reports related to pancreatic cancer increased. In newspaper articles, topics including new technologies related to pancreatic cancer and the related companies have significantly increased.

Our study has four implications. First, it is estimated that people's information exploration and information demands regarding pancreatic cancer have increased. The provision of information on pancreatic cancer is also increasing. Both questions related to pancreatic cancer in the Naver Q&A forum and articles related to pancreatic cancer in the media showed a growth trend. Assuming that the number of items or writings observed on the Internet and the media reflects people's interests, we interpret that the number of people seeking information online about deadly diseases, such as pancreatic cancer, has increased.

Second, appropriate information on pancreatic cancer is not enough in the Naver Q&A forum. The increase in mention of the upper back and lower back in questions concerning pancreatic cancer symptoms is a peculiar phenomenon because there are no medical grounds for the assumption that back pain is an early symptom of pancreatic cancer.

Although back pain can occur in 12–16% of patients diagnosed with pancreatic cancer, the positive predictive value (PPV) of pancreatic cancer among patients with back pain in primary clinics is less than 0.2%^54^. Hence, a very small proportion of patients with back pain in primary care had pancreatic cancer. Back pain is a very common symptom that people experience. Jaundice or abdominal pain with weight loss are more predictive symptoms of pancreatic cancer.^55^ Thus, if people pay too much attention to back pain and link the symptom to pancreatic cancer, then unnecessary anxiety and medical diagnosis could increase. It is difficult to learn exactly why mentioning back pain in symptom-related questions has increased; it may be related to patients with advanced-stage pancreatic cancer experiencing back pain. We can find that several respected institutions present back pain as an important symptom of pancreatic cancer^56^. If we read the information in detail, back pain is induced by a tumor pressing on the spine in the advanced stage. However, people might confuse one of the symptoms experienced by patients after significant progression of the disease with early symptoms. This trend demonstrates that there is a lack of adequate information available considering the increased interest.

We can identify similar implications from the results of classifying answers by two medical doctors, who are family medicine specialists and the authors of this paper. There was considerable misinformation and advertisements in the answers to the questions regarding pancreatic cancer symptoms. This indicates the need for access to adequate information.

Third, we suppose that the influence of a celebrity or public figure’s pancreatic cancer experience on the public interest in pancreatic cancer depends on how familiar the figures are to the public. Previous studies have found that the diagnosis of pancreatic cancer by public figures tends to increase social interest^23^. In our news analysis, many topics were related to various celebrities’ pancreatic cancer experiences. However, only topic clusters on Korean celebrities and sports stars were positively associated in proportion with topic clusters on pancreatic cancer symptoms and tests observed in Naver’s questions. We can hypothesize that not all celebrities’ pancreatic cancer experiences stimulate public interest, only those of familiar celebrities who are easy to sympathize with. However, our results are only a correlation in proportion change, so we are not sure about the direction of influence between the two factors: celebrity news and public interest.

Fourth, news articles related to new technologies for diagnosing and treating cancer, including pancreatic cancer, and related companies may be linked to public interest in pancreatic cancer. The relative proportion of the new technologies’ topic cluster (news cluster 2) related to pancreatic cancer increased the overall media coverage. A large proportion of the topics in the cluster are about new technologies for pancreatic cancer diagnosis and treatment. Simultaneously, the proportion of the clusters on pancreatic cancer diagnostic tests and symptoms (question clusters 3 and 4) from the Naver Q&A forum increased overall. The proportion change in the test-related topic cluster and symptom-related topic cluster from the Naver Q&A forum and the proportion change in the pancreatic cancer-related new technologies topic cluster from official media articles are estimated to be positively correlated.

Additionally, we suppose that public interest in biomarker tests using blood is high because media reports on new technology have raised people's general interest. In news cluster 2, there are a total of two topics explicitly related to diagnosing test techniques (Topics 12 and 66). Both topics deal with blood-based tests, indicating that blood biomarker tests are more frequently exposed to mass media than other test techniques. We speculate that the continuous increase in blood test keywords observed in the Naver Q&A forum may be related to this unbalanced media exposure.

A more rigorous analysis is needed to ensure the association between news articles related to the new technologies of various biotechnology companies and the public interest in pancreatic cancer. Our result shows a correlation between the proportion change of topic clusters extracted from media articles and diagnostic test-related topic clusters or symptom-related topic clusters extracted from the Naver Q&A forum. However, the hypothesis that various new technologies have attracted attention and increased public interest in related testing techniques and symptoms is worthwhile to be tested.

Pancreatic cancer is a deadly and rare disease, and the more fatal the disease is, the more desperate and frightened the patients and their acquaintances are. Desperation and fear are prime conditions for rumors and misinformation that affect people^10–12^. The uncertain possibilities of new technologies that attract people's attention could also increase under these conditions. Rare diseases hardly receive significant social attention. Hence, there might not be enough appropriate information on the disease and verification of the activities surrounding it for pre-existing information^15,16^. These are also good conditions for the spread of misinformation. In summary, pancreatic cancer has good conditions for rumors and misinformation to affect people. Therefore, monitoring online information exchange surrounding pancreatic cancer is a necessary task, and this study has attempted to do so. Government and health authorities should strive to provide adequate information about pancreatic cancer on the Internet, prevent the effects of existing misinformation, and continue to monitor the trends in online medical information exchanges in the future. Our research provides an excellent reference for these activities.

This study has some notable points in the data and methodology compared to other studies. First, our study analyzed long-term Internet language data for a rare but fatal disease, pancreatic cancer. Existing studies that monitor online information exchange mainly deal with diseases that generate great public interest over a short period (e.g., large-scale epidemics)^17–20^. However, the online information exchange on deadly diseases, which has long created fear, also significantly affects people's judgment and behavior and may have a more decisive impact than short-term pandemics because information verification is relatively inactive in this domain. Research in these fields has been rare considering their necessity, so our work dealing with this domain has great significance.

Second, this study identified the characteristics of information and communication through computer-based approaches. This approach extends the scale of the research data and allows for the relative objectivity of researchers’ judgment. Previous research on online information exchange has often been based on a small amount of data^57–59^, and human interpretation is often necessary to assess the characteristics and appropriateness of the information, although data should be limited to human performance. Recently, research on analyzing large amounts of language data using text mining has been actively introduced in health science^60–63^. We also used STM and keyword network analysis to exploit the advantages of this type of computer-based language data analysis. Our study could serve as a reference for research applying similar methods to large amounts of language data in the future.

Third, it is noteworthy that we extracted topic clusters based on the correlation between topics, rather than human reading. In other words, this work presents a method for summarizing the results of topic-modeling methods. When researchers use topic modeling methods, they frequently face problems stemming from a large number of topics (e.g., 100 topics from 50,000 documents). To grasp the structure of topics and subjects in an entire dataset, researchers must summarize the estimated topics. Therefore, our method, which utilizes a network community detection algorithm, can be a useful reference point.

This study has some limitations. First, the data reflecting public interest utilized by our research are from a single source, the Naver Q&A forum. However, it is practically impossible to collect all Internet data, which is an inevitable limitation shared by most studies utilizing them. Second, our results do not reflect the interest of the entire Korean population because the users of Naver or news articles cannot represent the entire population. Considering that our research aims to analyze the online information environment, we believe this limitation is inevitable. Third, proving whether the proportion of topics or topic clusters is proportionally associated with other topics or topic clusters cannot be claimed by simple correlations; thus, in this regard, this study can only produce probable hypotheses. Fourth, the simple regression model and Pearson’s correlation coefficient only express the linear association between variables. In future research, methods for the non-linear pattern are required.

However, our work explores the Internet information structure regarding a fatal but rare disease, pancreatic cancer, utilizing long-term data. We also introduce statistical methods based on large-scale data to delve into this area. Considering the importance of exploring online information exchange on deadly diseases, other forms of data collection and analysis, such as a survey, will also be required in future research.

## Supplementary Information

Supplementary Information.

## Data Availability

The data are publicly available at https://kin.naver.com/ (Naver Q&A forum / Naver questions) and https://www.kinds.or.kr/ (BIGKinds / news articles). The datasets gathered by us during the current study are available from the corresponding author upon reasonable request.
