# Supplementary material for: Online information analysis on pancreatic cancer in Korea using structural topic model
Source: Sci Rep. 2022 Jun 23;12:10622. doi: 10.1038/s41598-022-14506-1 (PMC9218710; doi:10.1038/s41598-022-14506-1)

Online information analysis on pancreatic cancer in Korea using structural topic model

Wonkwang Jo^1,4^, Yeol Kim^2*^, Minji Seo^2+^, Nayoung Lee^2+^, Junli Park^3+^

^1^ Department of Public Health Sciences, Graduate School of Public Health, Seoul National University, Seoul, Republic of Korea

^2^ National Cancer Control Institute, National Cancer Center, Goyang, Republic of Korea

^3^ Department of Family Medicine, Myongji Hospital, Goyang, Republic of Korea

^4^ Institute of Health and Environment, Seoul National University, Seoul, Republic of Korea

*Corresponding Author: Yeol Kim/ Corresponding author email: drheat@ncc.re.kr

^+^ these authors contributed equally to this work

**Supplementary Information**

Supplementary table S1. A list of media sources

| Media source name | number of articles |
| --- | --- |
| KBS | 61 |
| MBC | 163 |
| SBS | 202 |
| Maeil Business Newspaper | 1373 |
| Money Today | 840 |
| The Seoul Economic Daily | 667 |
| The Financial News | 536 |
| The Korea Economic Daily | 891 |
| The Herald Business | 652 |
| The Kyunghyang Shinmun | 405 |
| The Kukmin Daily | 693 |
| The Naeil shinmun | 111 |
| The Munhwa llbo | 217 |
| The Seoul Shinmun | 448 |
| The Segye Times | 544 |
| The Hankyoreh | 303 |
| The Hankook Ilbo | 389 |

Supplementary table S2. Interpretation and clusters of 53 topics from Naver’s questions

| **topic** | **interpretation** | **C** | **Top words by probability** | **Top words by FREX** |
| --- | --- | --- | --- | --- |
| 6 | Treatment and prognosis after diagnosis of pancreatic cancer | 1 | diagnosis, method, terminal stage | terminal stage, method, survival |
| 8 | How to help treat pancreatic cancer (including folk remedies) | 1 | pancreatic cancer, treatment, anti-cancer treatment | cure, complete recovery, mushroom |
| 9 | Diagnosis and treatment of pancreatic cancer and choice of hospital | 1 | hospital, university, medicine | hospital, university, neighborhood |
| 12 | Pancreatic cancer initial symptoms and prevention | 1 | symptom, food, early | food, early, care |
| 13 | Pancreatic cancer insurance money | 1 | insurance, insurance payment, pancreatic cancer | apply, notice, claim |
| 15 | Stories including Steve Jobs and pancreatic cancer. | 1 | Jobs, Apple, friend | Jobs, Apple, iPad |
| 18 | Worried about pancreatic cancer through family and various events | 1 | mother, father, words | mother, father, uncle |
| 25 | Treatment for pancreatic cancer | 1 | anticancer, treatment, radiation | anticancer drugs, radiation, remedy |
| 26 | A story of one’s dream. | 1 | dream, words, study | dream, dream reading, study |
| 29 | Medical accidents, negligence, lawsuits. | 1 | after, hospitalization, ambulatory care | Seoul Hospital, Samsung hospital, lawsuit, veterans hospital |
| 30 | Pancreatic cancer and human stories | 1 | illness, human, thought | story, disease, Korea |
| 31 | How to deal with end-stage pancreatic cancer | 1 | doctor, condition, words | words, doctor, situation |
| 36 | various incidents in the hospital | 1 | hospital, check, after | weight, landlord, holidays |
| 39 | Grandparents’ pancreatic cancer | 1 | At the time, degree, grandmother | grandmother, grandfather, inheritance |
| 40 | Family care after a pancreatic cancer diagnosis | 1 | admission, meal, ascites | ascites, nutrition supplements, recuperation |
| 43 | Family members’ struggle with pancreatic cancer | 1 | teeth, father, time | us, consciousness, resident |
| 44 | pancreatic cancer surgery | 1 | operation, recurrence, father | operation, recurrence, lymph node |
| 47 | Pancreatic Cancer Early Diagnosis Kit and Blood Test | 1 | song, early stage, relative | song, relative, early stage |
| 49 | Tragic personal history (including some of the pancreatic cancer stories) | 1 | father, home, money | marriage, asset, reception |
| 20 | A topic related to health insurance or cancer insurance | 2 | cancer, insurance, guarantee | expensive, small sum, product |
| 21 | A topic related to health insurance or cancer insurance | 2 | insurance, joining, cancer | breaking news, actual expense, popularity |
| 24 | A topic related to health insurance or cancer insurance | 2 | amount, 10,000 Korean won, joining | amount, breakwater, limit |
| 33 | A topic related to health insurance or cancer insurance | 2 | lead, expiration, 20 years | 20 years, payment, expiration |
| 38 | A topic related to health insurance or cancer insurance | 2 | diagnosis, cancer, injury | definite, being the first, opening day |
| 45 | A topic related to health insurance or cancer insurance | 2 | 10,000 Korean won, injury, illness | diagnostic fee, operation expenses, 10 |
| 52 | A topic related to health insurance or cancer insurance | 2 | payments, cancer, insurance | disaster, disability, payment |
| 2 | Blood tests and pancreatic cancer. | 3 | figure, blood test, normal | figure, blood test, normal |
| 3 | Questions regarding pancreatic abnormalities | 3 | pancreas, cognition, size | pancreas, centimeter, cognition |
| 4 | Questions regarding suspected pancreatic cancer findings and diagnostic tests | 3 | test, result, tissue | test, result, tissue |
| 5 | Health check-up and pancreatic cancer concerns. | 3 | checkup, health, comprehensive | checkup, health, comprehensive |
| 7 | Early screening of high-risk cancers, including pancreatic cancer. | 3 | cancer, case, patient | cancer, gene, danger |
| 11 | major cancer screening | 3 | stomach cancer, liver cancer, colon cancer | liver cancer, stomach cancer, colon cancer |
| 17 | Pancreatic cancer concerns related to lumps and other diseases. | 3 | bump, water, tumor | bump, CT, water |
| 23 | Suspected pancreatitis and pancreatic cancer | 3 | pancreatitis, hospitalization, week | pancreatitis, week, next |
| 35 | Pancreatic cancer symptoms and diagnostic methods | 3 | under, body, biliary stone | egg, biliary stone, stone |
| 37 | Concern about pancreatic cancer related to CA19-9 and blood test results. | 3 | gall bladder, doctor, degree | vesica, CA19-9, tumor marker |
| 42 | Diabetes and pancreatic cancer | 3 | diabetes, blood sugar, sugar | diabetes, blood sugar, insulin |
| 48 | Comprehensive medical examination results or examination items | 3 | test, illness, function | hearing ability, hepatitis, erythrocyte |
| 50 | Pancreatic cancer diagnosis and abdominal CT. | 3 | CT, ultrasound, abnormality | CT, ultrasound, MRI |
| 53 | Pancreatic cancer levels and how to respond after a family member is diagnosed with pancreatic cancer. | 3 | before month, reference | this time, past, reference |
| 1 | Worried about pancreatic cancer starting from indigestion and digestive abnormalities; | 4 | symptom, digestion, epigastrium | digestion, dyspepsia, epigastrium |
| 10 | Abdominal abnormalities and suspected pancreatic cancer (focusing on bowel movements and gas chams) | 4 | stomach, gas, colon | gas, enteritis, abdomen |
| 14 | Blood in stool and suspected pancreatic cancer | 4 | blood, words, hematochezia | bloody stool, blood, hemorrhoid |
| 19 | Back abdominal pain and pancreatic cancer | 4 | ache, back, waist | ache, back, flank |
| 22 | Jaundice, abdominal pain, weight loss and pancreatic cancer. | 4 | pancreatic cancer, worry, jaundice | jaundice, worry, abdominal pain |
| 27 | Change in bowel movements and suspected pancreatic cancer | 4 | excreta, diarrhea, symptom | grey, mutation, stool |
| 32 | Risk of pancreatic cancer related to smoking | 4 | alcohol, cigarette, smoking | alcohol, pack, sputum |
| 34 | Stress, anxiety, pancreatic cancer. | 4 | degree, stress, symptom | hypochondriasis, bacteria, anxiety |
| 46 | Worrying about various symptoms such as weight loss and pancreatic cancer; | 4 | emergency room, stomach, medicine | emergency room, herbal medicine clinic, calculus |
| 16 | Obesity, weight, and cancer risks | 5 | obesity, ingestion, weight | obesity, calorie, sugar |
| 28 | Smoking and cancer | 6 | tobacco, smoking, nicotine | motion sickness, nicotine, second-hand smoking |
| 41 | Questions regarding suspected pancreatic cancer symptoms and diagnosis of pancreatic cancer | 7 | occur, construction, part | erection, styrofoam, occupancy |
| 51 | Occupational and pancreatic cancer insurance questions | 8 | company, occupation, diagnosis | job, 3 days, work |

Supplementary table S3. Summary of simple linear regression models (topics in cluster 4)

| Summary of simple linear regression models  Response variable: topic proportion per year  Explanatory variable: year | | | |
| --- | --- | --- | --- |
| Topic number | Year’s coefficient | Std. Error | *P* value |
| 1 | 0.002145 | 0.00049 | < 0.001 |
| 10 | 0.000729 | 0.000306 | 0.030 |
| 14 | -0.000829 | 0.000374 | 0.041 |
| 19 | 0.002607 | 0.000356 | < 0.001 |
| 22 | 0.001379 | 0.000582 | 0.031 |
| 27 | 0.001092 | 0.000211 | < 0.001 |
| 32 | 0.000002 | 0.000139 | 0.989 |
| 34 | 0.002111 | 0.00045 | < 0.001 |
| 46 | 0.001062 | 0.000245 | < 0.001 |

Supplementary table S4. Summary of simple linear regression models (topics in cluster 3)

| Summary of simple linear regression models  Response variable: topic proportion per year  Explanatory variable: year | | | |
| --- | --- | --- | --- |
| Topic number | Year’s coefficient | Std. Error | *P* value |
| 2 | 0.000345 | 0.000106 | 0.005 |
| 3 | -0.000048 | 0.000103 | 0.647 |
| 4 | 0.000135 | 0.000136 | 0.335 |
| 5 | 0.000589 | 0.000139 | < 0.001 |
| 7 | -0.00013 | 0.00015 | 0.400 |
| 11 | 0.000403 | 0.000174 | 0.034 |
| 17 | 0.000235 | 0.000176 | 0.202 |
| 23 | -0.000181 | 0.000261 | 0.499 |
| 35 | -0.000476 | 0.00015 | 0.006 |
| 37 | -0.000023 | 0.000259 | 0.930 |
| 42 | 0.000569 | 0.000254 | 0.039 |
| 48 | 0.000456 | 0.000286 | 0.131 |
| 50 | 0.001151 | 0.000156 | < 0.001 |
| 53 | -0.000202 | 0.000063 | 0.006 |

Supplementary table S5. Interpretation and clusters of 75 topics from news articles

| **topic** | **interpretation** | **C** | **Top words by Probability** | **Top words by FREX** |
| --- | --- | --- | --- | --- |
| 5 | Steve Jobs and pancreatic cancer | 1 | Jobs, report, health | Enquirer, a tabloid, sick leave |
| 10 | Romance drama and pancreatic cancer | 1 | life, death, oneself | Stay, Oh Jung-se, character type |
| 21 | Research achievements of major universities including UNIST | 1 | UNIST, university, professor | Andraka, UNIST, sea water battery |
| 27 | Steve Jobs and Apple | 1 | Jobs, Apple, Steve Jobs | Isaacson, Chief Operating Officer, respiratory standstill |
| 37 | A character in a drama and pancreatic cancer | 1 | drama, acting, appearance | Kim Jae-won, Jang Dae-hee, class |
| 44 | Literary works and pancreatic cancer | 1 | Writer, story, novel | Jeong Jin-ki, Standard Chartered, programming |
| 6 | Biotechonology companies and investment markets | 2 | bio, investment, corporation | AptaBio, offering price, OCI |
| 12 | Patent for early diagnosis of pancreatic cancer based on blood and related companies | 2 | diagnosis, pancreatic cancer, early | JW Holdings, JW bioscience, diagnositic kits |
| 20 | Bio companies’ launching of new products and entering the market | 2 | market, bio, president | SamYang Genex Corporation, urine chemistry, exosome |
| 25 | Boryeong Pharmaceutical’s Medical Literature Award and SK Hynix’s occupational disease compensation | 2 | target, brand, Boryung Pharmaceutical Co. Ltd. | Boryung Pharmaceutical Co. Ltd, occupational disease, inspection commission |
| 28 | New pancreatic cancer treatment and pancreatic cancer in a drama | 2 | Pancreatic cancer, GV1001, result | softening, white pebble, JP |
| 29 | Pharmaceutical companies’ new drug technology and patents | 2 | material, pharmaceutical company, Cellivery Co. | salivary, TSDT, Wnt |
| 31 | Genes and cancers (such as breast cancer) | 2 | gene, breast cancer, protein | Ras, BRCA1, BRCA2 |
| 41 | Development, patenting, and approval of new pancreatic cancer treatments | 2 | treatment, pancreatic cancer, treatment | C&Farm, optical fiber, Polytaxel |
| 45 | Investment opportunities in companies related to the treatment of various diseases, including pancreatic cancer | 2 | business, representative, company | browser, OncoQuest Pharmaceuticals Inc., convertible bond |
| 52 | News about new anticancer drugs | 2 | clinical, patient, anti cancer drug | Suten, Nexavar, Fenbendazole |
| 55 | Securities investment proposal | 2 | event, material, soaring | SanseongPNC, top-secret, 0% |
| 56 | Corporate information related to clinical trials for new anticancer drugs | 2 | Gembax, clinical, GV1001 | anti-cancervaccine, purchase volume, Gemvax |
| 61 | New cancer treatments such as treatments using dendritic cells | 2 | cell, treatment, cancer cell | cancer antigen, CD8-T cell, medical association |
| 64 | Stock market and stock price changes | 2 | stock, increase, decrease | net selling, industrial classification, downtrend |
| 66 | New technologies for NK cell measurement and related cancer treatments | 2 | NK, cell, examination | ATIGEN, NK View Kit, NK Max |
| 68 | Various approvals for new anticancer drugs (clinical test approval, rare drug designation approval) and news regarding related companies | 2 | clinical, remedy, anticancer drugs | HeimBio, GC Green Cross Cell, CG200745 |
| 72 | Genetic testing and prevention of diseases including cancer | 2 | gene, test, genomic | gluten, genomic, HT |
| 73 | A major North Korean figure’s death by pancreatic cancer | 2 | chairperson, North Korea, Kim Jong-il | Chen Ziming, Yon Hyong-muk, national defense committee (NDC) |
| 75 | Patents related to GV1001 and its anticancer effect and news of related companies | 2 | effect, anticancer, patent | application, effect, anticancer drug |
| 1 | Eating habits and cancer | 3 | intake, danger, obesity | processed meat, BMI, obesity |
| 2 | Introduction of new cancer treatment equipment | 3 | cure, radiation, tumor | HIFU, cyber knife, hype |
| 7 | AI-based diagnosis and new cancer treatment methods | 3 | patient, treatment, doctor | SB injection, Watson, Radiological technologists |
| 11 | Pancreatic cancer symptoms and surgery | 3 | pancreatic cancer, pancreas, diabetes mellitus | pancreatitis, chronic relapsing pancreatitis, pancreas |
| 23 | Necessity and effectiveness of various diagnostic tests, including health check-ups | 3 | test, checkup, detect | health screenings, colon fiberscope, checkup |
| 39 | News of famous professors and cancer experts and health lectures | 3 | Professor, pancreatic cancer, held | lecture on health, Euless, stent |
| 40 | Opening treatment institutions such as cancer centers and introducing equipment | 3 | treatment, Cancer Center, baryon | baryon accelerator, baryon, accelerator |
| 47 | Symptoms and preventive measures for various diseases | 3 | disease, symptoms, health | spinach, vitamin, backache |
| 48 | Smoking and cancer | 3 | smoking, tobacco, stop smoking | a piece of splint, cigarette, withdrawal |
| 51 | Euthanasia and death of a patient | 3 | patient, euthanasia, victim | Choi Cheol-soo, Han Youn-seo, Dignitas |
| 59 | Soldiers’ discharge due to cancer and pancreatic cancer | 3 | hospital, medical care, treatment | captain, Ro Chung-guk, soldier |
| 62 | Information related to chemotherapy | 3 | treatment, patient, patients | Samsung cancer hospital, chemotherapy, Nexia |
| 67 | New cancer surgery methods such as laparoscopic surgery | 3 | surgery, professor, patient | Robotic surgery, classification, laparoscopic |
| 4 | Pancreatic cancer of a Korean celebrity family | 4 | photo, husband, pancreatic cancer | Choi Ji-woo, Lee Hye-kyung, Lee Hwa-seon |
| 17 | Korean soccer coach Yoo Sangchul’s fight with pancreatic cancer | 4 | director, Incheon, Yoo Sang-Chul | Yoo Sang-cheol, K-League, winning point |
| 50 | A citizen who appeared on a TV show and pancreatic cancer | 4 | broadcast, program, appearance | Voice Queen, Park Chan-ho, Lee Yeon-bok |
| 13 | Korean celebrity’s death by pancreatic cancer | 5 | actor, human, Kim Young-ae | Sunshine, Kim Sang-yeol, Kim Ja-ok |
| 33 | Korean actress Kim Young-ae’s death by pancreatic cancer | 5 | Kim Young-ae, acting, pancreatic cancer | Red clay pack, The gentlemen of wolgyesu tailor shop (Korean drama), Lee Young-don |
| 58 | Daily life stories related to family members, including celebrities’ families | 5 | father, Buddhist monk, Kim Min-gyo | Kim Min-kyo, Whee-In, MAMAMOO |
| 24 | The burden of medical expenses and health insurance | 6 | insurance, patient, health | Abraxane, guarantee rate, no-show |
| 26 | Statistical information on major cancers | 6 | survival, patient, stomach cancer | cancer patient, 2001~2005, 100.2% |
| 38 | Death statistics | 6 | dead, death, suicide | transport accident, crude death rate, 16.5 persons |
| 9 | Cheonghae unit member’s family's death by pancreatic cancer | 7 | death, last, father | Rachel, Lee Hwan-wok, Cheonghae Unit (Korean vessels) |
| 15 | Various stories on cancer in the family | 7 | husband, wife, (married) couple | Park In-soo, Gak Bok-hwa, Shin Jin-goo |
| 60 | Litigation and legal dispute | 7 | lawsuit, accident, suspicion | Yoon Chang-joong, industrial hazards, death in the line of duty |
| 32 | A family story that appears in novels and introduction of a famous person | 8 | mother, father, mother | Margaret, Dennis, Carroll |
| 36 | Various stories about people's lives | 8 | human, people, god | Huh Kyung-young, attention, conduction |
| 65 | Writers and pancreatic cancer | 9 | poet, Kim Nam-joo, literary | Kim Nam-joo, Go joung-hi, Nam min-jeon |
| 71 | Death of famous Korean people who are not celebrities by pancreatic cancer | 9 | deceased, pass away, region | Elbow society, Iljisa, Son Myeong-jo |
| 3 | Pancreatic cancer among Chinese politicians | 10 | China, deputy prime minister, Huang Ju | Huang Ju, Shanghai clique, Chinese parliament |
| 8 | Patrick Swage’s death from pancreatic cancer | 11 | movie, director, Patrick | Hurt, Dirty Dancing, Snow piercer |
| 14 | Teeth and health | 12 | gum, tooth, disease | gum, implant, gingiva disease |
| 16 | Pancreatic cancer death of a chairman of a major company or ministry of government | 13 | chairman, minister, economy | Kang Bong-gyun, the Ministry of Finance and Economy, Sung woo |
| 18 | Newspaper serial novels or daily life stories regarding pancreatic cancer | 14 | woman, male, human | Kim Seung min, Kim Nam-sook, Kwang-jo bank |
| 19 | Major Japanese Okinawa politician’s death by pancreatic cancer | 15 | Japan, Abe, Branch Office | Okinawa, Okinawa, the Liberal Democratic Party |
| 22 | Pancreatic cancer of major foreign politicians and bureaucrats | 16 | president, the United State, Ginzberg | Ginsburg, Mubarak, Lewinsky |
| 30 | Korean actress Jin Dohee’s death by pancreatic cancer | 17 | Jin Do-hee, pass away, pancreatic cancer | Jin Do-hee, chase, Kim tae-ya |
| 34 | Famous Korean-American Dr. Kang Youngwoo’s death by pancreatic cancer | 18 | doctor, disabled person, the United State | SeokEun-ok, Kang Young-woo, Rotary International |
| 35 | Crimes against women, cases regarding women | 19 | woman, the United State, Covid-19 | ride, Gyeongju Hospital, Sally |
| 42 | Deep Purple band member’s death by pancreatic cancer | 20 | road, music, band | purple, road movie, Keyboardist |
| 43 | Death of famous Korean people who are not celebrities by pancreatic cancer | 21 | deceased, pancreatic cancer, direct | Seoul National University of Arts, Jang Hyeongil, JoBio |
| 46 | Chanel designer Lagerfeld’s death by pancreatic cancer | 22 | Lagerfeld, chanel, fashion | Lagerfeld, Spat, Fendi |
| 49 | Educators’ fight with pancreatic cancer and death | 23 | study, Kim joo-seung, Kim | Jang Young-ja, Kim joo-seung, mother in law |
| 53 | Cancer outbreak in a specific region | 24 | town, factory, residents | polar bear, Jangjom village, tobacco |
| 54 | Cancer-related insurance | 25 | guarantee, insurance, insurance status | cancer insurance, special contract, diagnosis fee |
| 57 | Korean comedian Song Jungeun’s event for pancreatic cancer patient | 26 | Song Joon-geun, fater, precedence | Song Joon-geun, wedding anniversary, precedence |
| 63 | The characters in a drama and pancreatic cancer | 27 | Euljihaei, Jang Hwa-sa, lawyer | Euljihaei, Jang Hwa-sa, Kisan |
| 69 | Pancreatic cancer in the family of major sports players | 28 | victory, (the game of) go, travel gear | KimTae-sul, Ahn shin-ae, travel gear |
| 70 | Foreign celebrities’ death by pancreatic cancer | 29 | Franklin, singer, song | Aretha, Choi Bak-ho, Gospel |
| 74 | Singer Pavarotti’s death by pancreatic cancer | 30 | Pavarotti, opera, tenor | Domingo, José Maria Carreras, Placido |

Supplementary Figure S1. The number of questions related to pancreatic cancer in the Naver Q&A forum


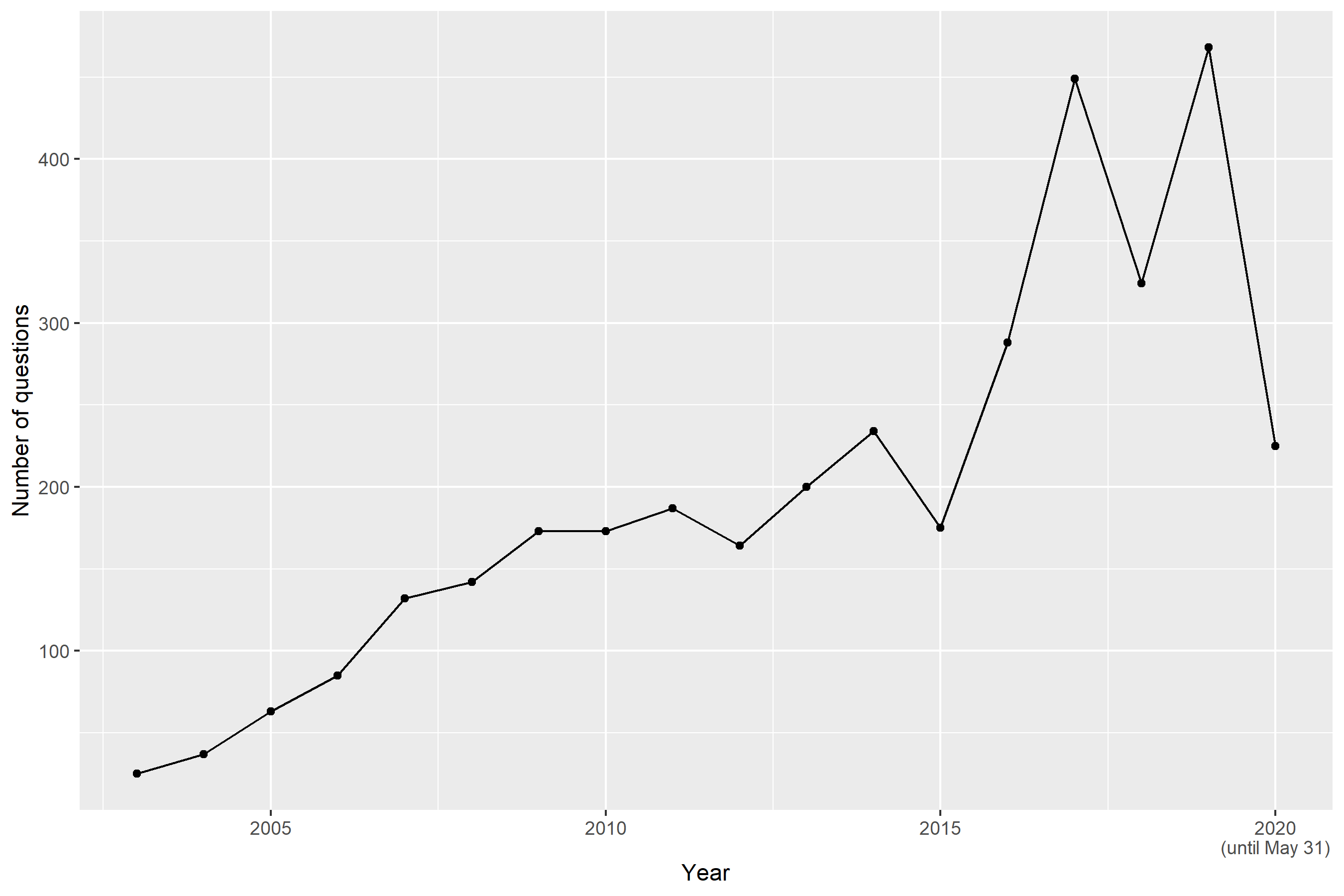


Supplementary Figure S2. Held-out likelihood of structural topic models from Naver’s questions


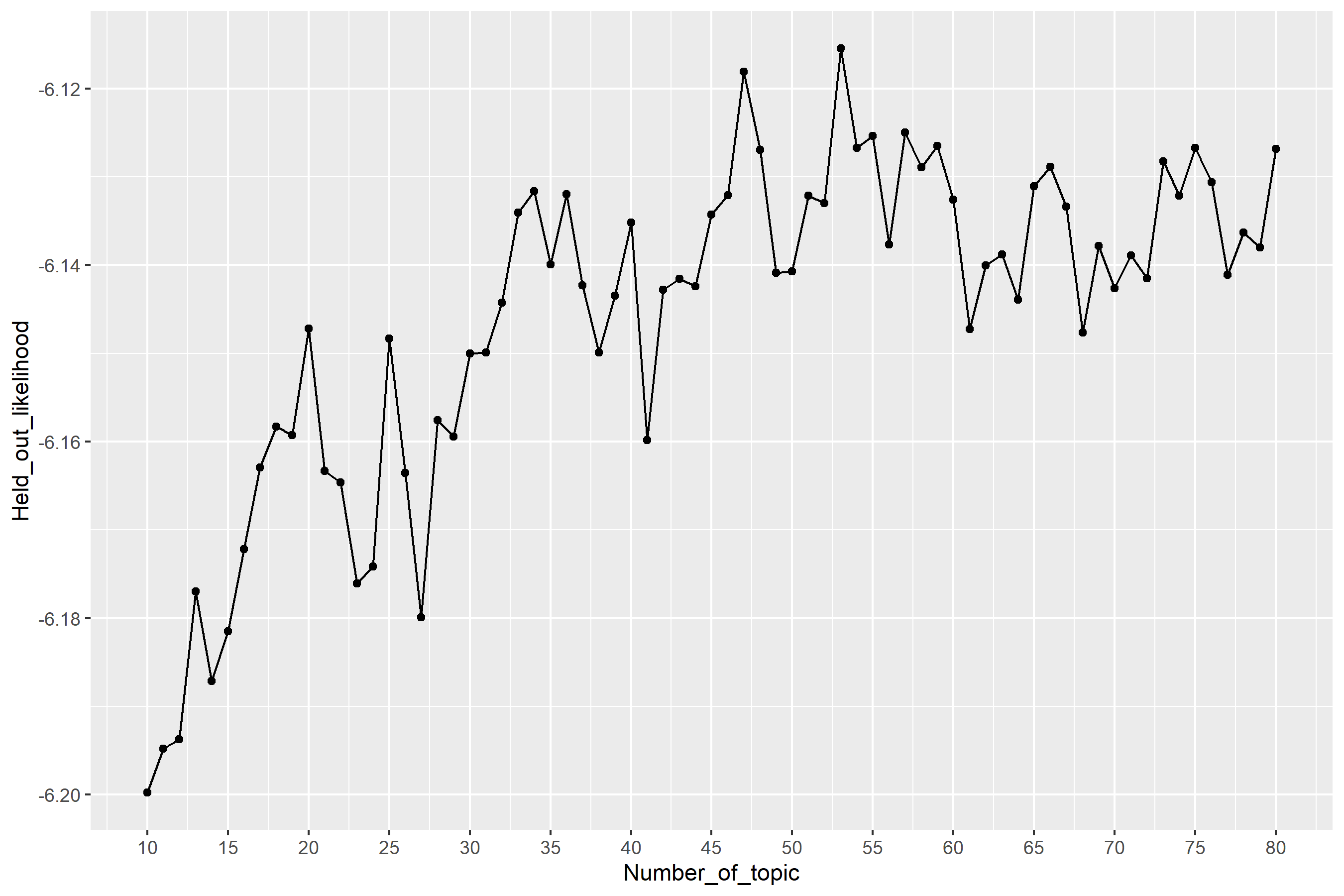


Supplementary Figure S3. Topic clusters visualization from 53 topics of Naver’s questions (layout: Fruchterman-Reingold)


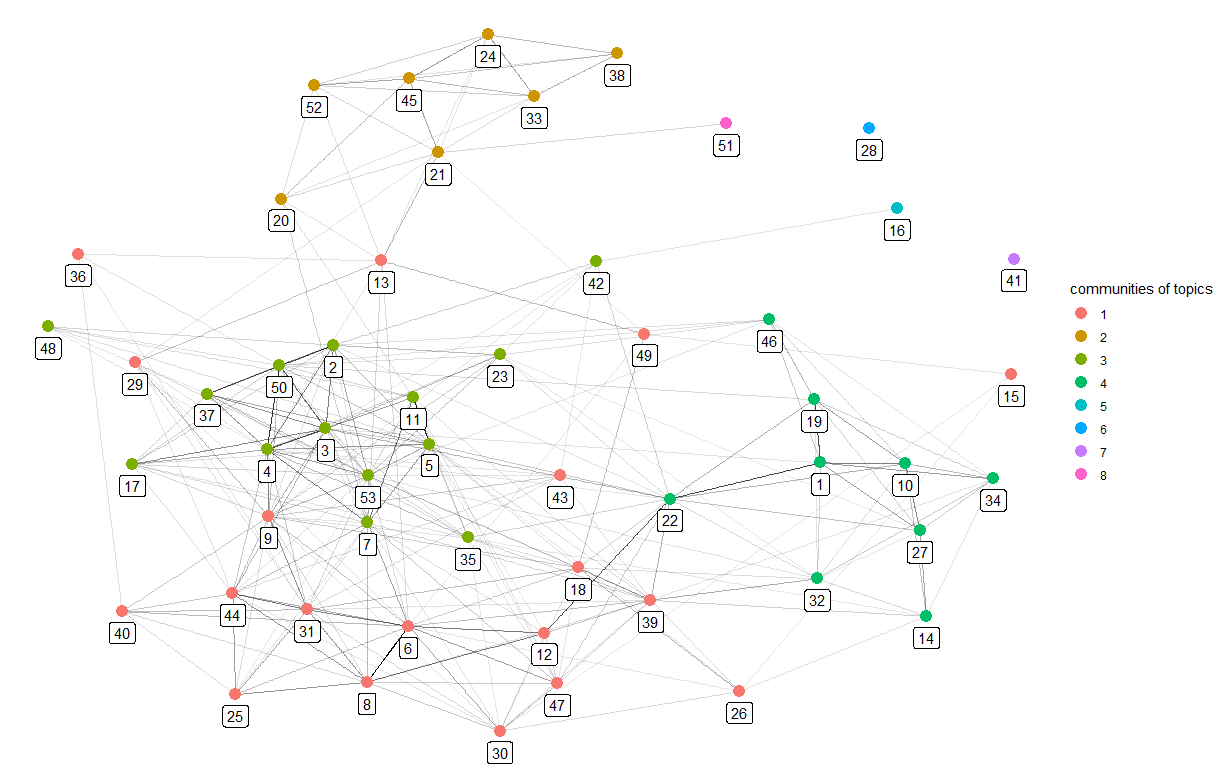


Supplementary Figure S4. The number of news articles related to pancreatic cancer by year


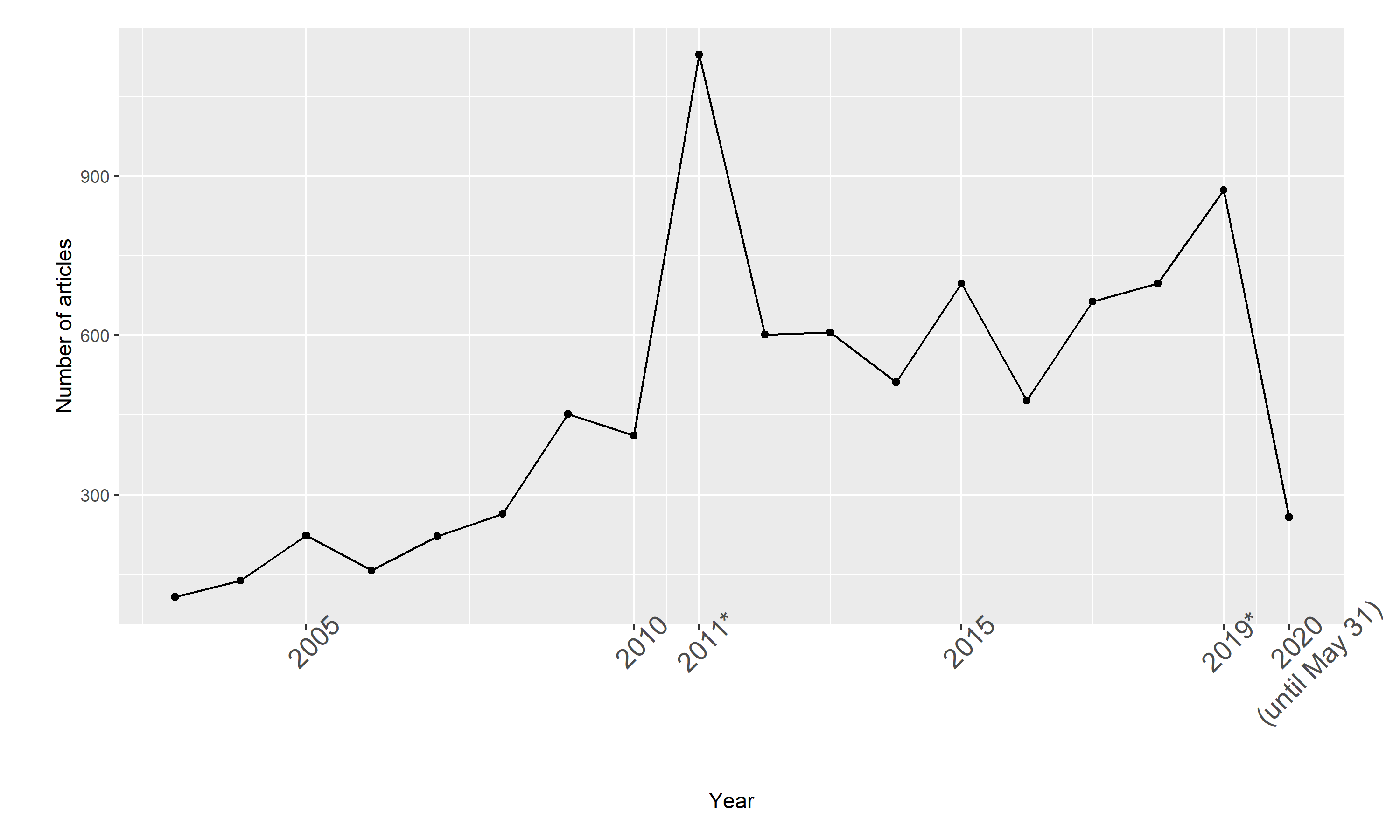


**Note: the death of Steve Jobs – 2011, Yoo Sang-Chul's diagnosis - 2019*

Supplementary Figure S5. Held-out likelihood of structural topic models from news articles


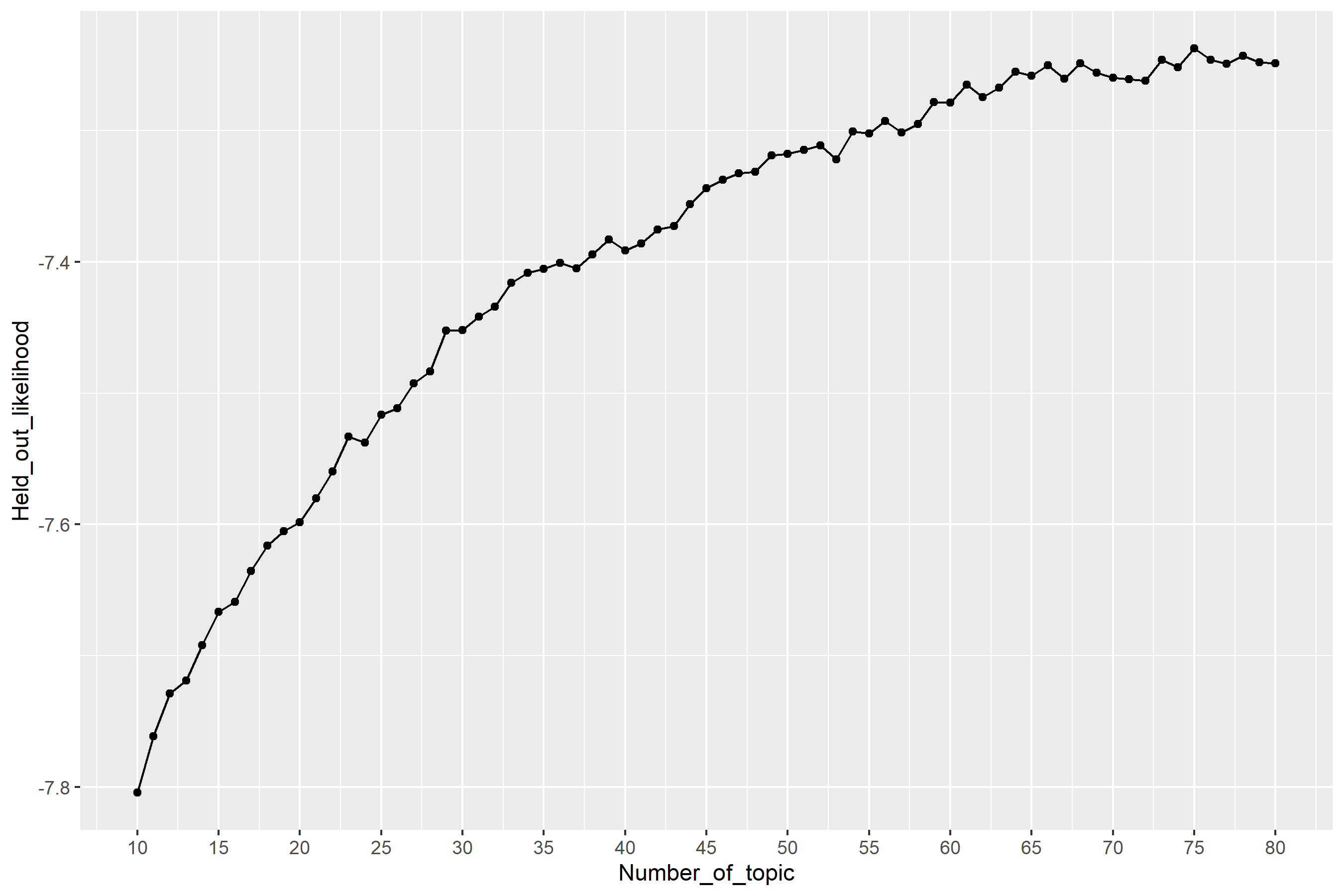

Supplement: Supplementary file 1 — Supplementary Information. [file 41598_2022_14506_MOESM1_ESM.docx]
